# Scalp Basal Cell Carcinoma Presenting as Alopecia

**DOI:** 10.5826/dpc.1101a129

**Published:** 2021-01-29

**Authors:** Alexandra C. Goetze, Giovana Liz Marioto de Campos, Felipe Bochnia Cerci, Betina Werner

**Affiliations:** 1 Clínica Newestetic, Curitiba, Brazil; 2 Department of Dermatology, Hospital Universitário Evangélico Mackenzie, Curitiba, Brazil; 3 Clínica Cepelle, Curitiba, Brazil; 4 Department of Dermatology, Hospital de Clínicas da Universidade Federal do Paraná, Curitiba, Brazil; 5 Department of Pathology, Hospital de Clínicas da Universidade Federal do Paraná, Curitiba, Brazil

**Keywords:** basal cell carcinoma, alopecic plaque, arborizing vessels, Mohs micrographic surgery, skin cancer

## Case Presentation

**Figure 1:**
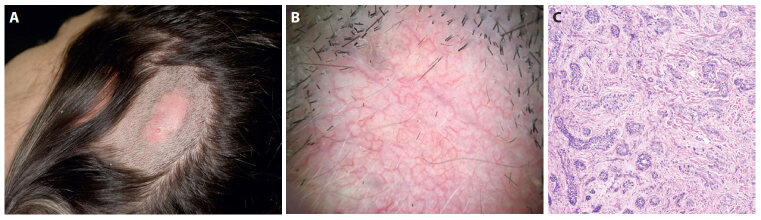
(A) Indurated alopecic plaque on the left parieto-occipital scalp. Trichotomy was performed around the plaque prior to the surgery. (B) Dermoscopy demonstrated multiple arboriform vessels. (C) Histopathology revealed infiltrative chords and irregular small nests of basaloid cells with palisading and clefting in some areas surrounded by a desmoplastic dermis. There was no sign of follicular germ or papilla formation throughout the neoplasia (H&E, original magnification ×10).

A healthy 32-year-old woman presented with a 7-year history of a slowly growing asymptomatic alopecia plaque on the scalp. Physical examination revealed a 4.7 × 2.4 cm erythematous indurated alopecic plaque on the left parieto-occipital scalp (Figure 1A). A 3 mm papule could be noticed on the inferior area of the plaque. Dermoscopy showed multiple arborizing vessels in the plaque with no follicular openings (Figure 1B). Histopathologic examination revealed an infiltrative basal cell carcinoma (BCC) with no evidence of sebaceous nevus (Figure 1C). The patient was treated with Mohs micrographic surgery followed by primary closure. At a 3-year follow-up, no recurrence was noted.

## Teaching Point

Alopecia of the scalp caused by BCC can manifest without pearly borders and ulceration and can be mistaken for other conditions such as discoid lupus erythematosus or alopecia areata [1]. An indurated plaque of alopecia with arborizing vessels on dermoscopy should raise the suspicion for BCC.
